# Supplementary material for: Propranolol: A “Pick and Roll” Team Player in Benign Tumors and Cancer Therapies
Source: J Clin Med. 2022 Aug 4;11(15):4539. doi: 10.3390/jcm11154539 (PMC9369479; doi:10.3390/jcm11154539)
Supplement: Supplementary file 1 [file jcm-11-04539-s001.zip › Table S1.pdf]

Table S1. **Propranolol treatment in clinical trials in Infantile Hemangioma disease**. Compilation of the interventional clinical trials registered at the EU Clinical Trials Register (https://www.clinicaltrialsregister.eu), the U.S. National Library of Medicine (https://clinicaltrials.gov), and the Australian New Zealand Clinical Trials Registry (http://www.anzctr.org.au/Default.aspx).
Acronyms: Status: C (Completed); NYR (Not yet recruiting); R (Recruiting); O (Ongoing); T (Terminated); U (Unknown). NoP (number of patients recruited) and NA (Not Applicable).

Trial ID	Study Title	Status	Conditions	Drugs and dosage	Phase	NoP	Start Date
NCT00744185	Propranolol in Capillary Hemangiomas	T	Hemangioma Capillary	Propranolol (3 - 4 mg/kg/d) Placebo (3 - 4 mg/kg/d)	2/3	14	2008
NCT01211080	Off Label Use of Propranolol for Infancy Hemangiomas	C	Hemangioma	Propranolol (2mg/kg/d)	NA	72	2008
ACTRN1261100 0004965	Propranolol for infantile haemangiomas	C	Infantile hemangioma	Propranolol (2 mg/kg/d) Placebo (2 mg/kg/d)	2	40	2009
NCT00967226	Propranolol Versus Prednisolone for Treatment of Symptomatic Hemangiomas	T	Hemangioma of Infancy	Propranolol (2 mg/kg/d) Prednisolone (2 mg/kg/d)	2	19	2009
NCT01056341	Study to Demonstrate the Efficacy and Safety of Propranolol Oral Solution in Infants With Proliferating Infantile Hemangiomas Requiring Systemic Therapy	C	Infantile Hemangioma	Propranolol (1 or 3 mg/kg/d) Placebo (Not provided)	2/3	512	2010
2009-014571-49	The use of systemic propranolol in congenital hemangiomas	O	Congenital Hemangioma	Not provided	2	10	2010
2009-018102-22	A multicentre, open-label, repeated-dose, pharmacokinetic study of Propranolol in infants treated for proliferating infantile hemangiomas (IHs) requiring systemic therapy	C	Infantile Hemangioma	Propranolol (1 - 3 mg/kg/d)	1	23	2010
2010-019754-41	Multicenter, prospective, single-arm phase III study of the efficacy and safety of oral therapy with propranolol (ProAngiol juice; 2 mg/kg bw/d with optional dose increase to 3 mg/kg bw/d) in infants 4	C	Hemangioma requiring systemic therapy	Propranolol (0.1 - 3 mg/kg/d)	3	66	2010

	weeks to 11 months of age with severe proliferating hemangiomas						
NCT01072045	Comparative Study of the Use of Beta Blocker and Oral Corticosteroid in the Treatment of Infantile Hemangioma	C	Hemangioma	Propranolol (2mg/kg/d)<br>Prednisone (2mg/kg/d)	2	50	2010
NCT01074437	A Phase II, Randomized, Double-Blind Comparison of Corticosteroid and Corticosteroids With Propranolol Treatment of Infantile Hemangiomas (IH)	T	Hemangioma	Propranolol (2mg/kg/d)<br>Prednisolone (1-2mg/kg/d)	2	9	2010
NCT04651049	Systemic Propranolol for the Treatment of Paediatric Patients With Infantile Hemangiomas	C	Infantile Hemangiomas	Propranolol (2mg/kg/d)	NA	128	2010
2011-003144-50	A randomised, double blind, controlled, multicentre study in infants with infantile hemangioma to compare propranolol gel to placebo	C	Infantile Hemangioma	Propranolol gel (150 mg/application)<br>Placebo (Not provided)	2	81	2011
NCT01512173	Study in Infants With Infantile Hemangioma to Compare Propranolol Gel to Placebo	C	Infantile Hemangioma	Not provided	2	82	2012
NCT01743885	Efficacy and Safety of Propranolol Versus Acebutolol on the Proliferative Phase of Infantile Hemangioma	T	Hemangioma	Propranolol (3 mg/kg/d)<br>Acebutolol (10 mg/kg/d)	3	55	2012
NCT01908972	The Safety and Efficiency of Propranolol as an Initial Treatment for Pediatric Hemangioma	C	Hemangioma	Propranolol (2mg/kg/d)<br>Prednisolone (2mg/kg/d)	4	34	2013
NCT02342275	Efficacy and Safety of Propranolol Versus Atenolol on the Proliferative Phase of Infantile Hemangioma	C	Hemangioma	Propranolol (1 - 2 mg/kg/d)<br>Atenolol (0.5 - 1 mg/kg/d)	3	377	2013
2014-005555-80	Efficacy and safety of Hemangiol solution in the treatment of high risk infantile hemangioma. A Multinational Single Arm Study	C	Infantile Hemangioma	Propranolol (1 - 3mg/kg/d)	3	45	2015
NCT02505971	Nadolol Versus Propranolol in Children With Infantile Hemangiomas	C	Infantile Hemangioma	Propranolol (0.5 - 1.5 mg/kg/d)<br>Nadolol (0.5 - 1.5 mg/kg/d)	3	74	2015
NCT03237637	Comparative Study to Evaluate the Effectiveness of Atenolol and Propranolol in the Treatment of Infantile Hemangiomas	U	Infantile Hemangioma	Propranolol (1 - 2mg/kg/d)<br>Atenolol (0.5 - 1mg/kg/d)	3	60	2017
NCT04105517	Hemangiol, Post Marketing Surveillance Study	C	Infantile Hemangioma	Propranolol (1 - 4 mg/kg/d)	NA	500	2019
NCT04288700	Evaluation of the Efficacy of Captopril Versus Propranolol and Timolol as a Treatment of Infantile Capillary Hemangioma	R	Infantile Hemangioma	Propranolol (2 mg/kg/d or 0.2mg intravitreal injection)<br>Captopril (0.45 - 0.9 mg/ kg/d)	4	100	2019

				Timolol maleate (3 eye drops/d)			
NCT04684667	Efficacy of Propranolol in the Treatment of Infantile Hemangioma	NYR	Infantile Hemangioma	Propranolol (0.5 - 2 mg/kg/d, 9)	2	100	2021
