# Supplementary material for: Propranolol: A “Pick and Roll” Team Player in Benign Tumors and Cancer Therapies
Source: J Clin Med. 2022 Aug 4;11(15):4539. doi: 10.3390/jcm11154539 (PMC9369479; doi:10.3390/jcm11154539)
Supplement: Supplementary file 1 [file jcm-11-04539-s001.zip › Table S2.pdf]

Table S2. **Propranolol in observational trials.** Compilation of the interventional clinical trials registered at the EU Clinical Trials Register (https://www.clinicaltrialsregister.eu), the U.S. National Library of Medicine (https://clinicaltrials.gov), and the Australian New Zealand Clinical Trials Registry (http://www.anzctr.org.au/Default.aspx). Status: C (Completed). NoP means number of patients recruited in the trial.

Trial ID	Study Title	Status	Conditions	Compared/ combined with	Outcome Measures	Drugs and dosage	Phase	NoP	Start Date	Results
NCT01211080	Off Label Use of Propranolol for Infancy Hemangiomas	C	Hemangioma	-	Cosmesis of the lesion and surrounding skin<br>Side effects<br>hemangioma size	Propranolol (2mg/kg/d)	O	72	2008	-
NCT04651049	Systemic Propranolol for the Treatment of Paediatric Patients With Infantile Hemangiomas	C	Infantile Haemangiomas	-	Change on the height of the patients.<br>Change on the weight of the patients	Propranolol (2mg/kg/d)	O	128	2010	-
NCT02165683	Use of Propranolol to Reduce FDG Uptake in Brown Adipose Tissue in Pediatric Cancer Patients PET Scans	C	Pediatric Cancer	-	Number of Adverse Events following Propranolol dosing.<br>Number of Reductions in FDG Uptake in BATWhen Compared to Historic Controls	Not provided	O	10	2014	-
NCT02334930	Evaluation of New Biomarkers Predictive of Efficacy Betablockers in PEComa and Vascular Pediatric Tumors	C	PEComa Hemangioma	-	YAP cellular expression by immunohistochemistry study on collected tissues<br>Beta adrenergic receptors expression by immunohistochemistry study on collected tissues<br>Study of Hippo/YAP pathway expression by western-blot, and correlation with propranolol efficacy<br>Study of MAP/kinases pathway expression by western-blot, and correlation with propranolol efficacy<br>Study of PKA/AMPc pathway expression by western-blot, and correlation with propranolol efficacy	Not provided	O	13	2015	-
NCT04105517	Hemangiol, Post Marketing Surveillance Study	C	Infantile Hemangioma	-	Incidence of adverse events in children with proliferative infantile hemangiomas receiving Hemangiol<br>Incidence of the regression of proliferative infantile hemangiomas in children receiving Hemangiol<br>Incidence of diagnosed heart diseases with systematic cardiac consultation events in children with proliferative infantile hemangiomas receiving Hemangiol	Propranolol (3-5 mg/kg/d)	O	500	2019	-
