# Supplementary material for: Propranolol: A “Pick and Roll” Team Player in Benign Tumors and Cancer Therapies
Source: J Clin Med. 2022 Aug 4;11(15):4539. doi: 10.3390/jcm11154539 (PMC9369479; doi:10.3390/jcm11154539)
Supplement: Supplementary file 1 [file jcm-11-04539-s001.zip › Table S3.pdf]

Table S3. P**ropranolol as adjuvant therapy at single administration or combinatorial treatment clinical trials.** Compilation of the interventional clinical trials registered at the EU Clinical Trials Register (https://www.clinicaltrialsregister.eu), the U.S. National Library of Medicine (https://clinicaltrials.gov), and the Australian New Zealand Clinical Trials Registry (http://www.anzctr.org.au/Default.aspx). Only interventional trials (with results or not finished), where propranolol was tested, are listed.
Phase: NA (Not Applicable). Status: C (Completed); NYR (Not yet recruiting); ANYR (Active, Not recruiting); R (Recruiting); O (Ongoing); T (Terminated); S (Suspended); U (Unknown); W (withdrawn). NoP means number of patients recruited in the trial.

Trial ID	Study Title	Status	Conditions	Compared/ combined with	Outcome Measures	Drugs and dosage	Phase	NoP	Start Date	Results
NCT01451658	A Trial of EVL\GVS Alone vs. EVL\GVS Combined Propranolol (S-HCC)	R	Gastroesophageal Varices Hemorrhage Hepatocellular Carcinoma	-	Rebleeding. Complication survival	Propranolol (20mg/d) titrated to decrease heart rate more than 25 % of baseline	4	100	2009	-
NCT01970748	Primary Prevention of Patients With Hepatocellular Carcinoma and Concomitant Esophageal Varices	R	Bleeding Esophageal Varices Hepatocellular Carcinoma	-	Rebleeding. Complication survival	Propranolol (20mg/d) titrated to decrease heart rate more than 25 % of baseline	4	200	2009	-
NCT01298284	A Trial of EVL\GVS Alone vs. EVL\GVS Combined Propranolol	U	Liver Cirrhosis Hepatoma	-	Rebleeding. Complication survival	Propranolol (20mg/d) titrated to decrease heart rate more than 25 % of baseline	4	60	2009	-
NCT00888797	Î²-adrenergic Blocker and a COX2 Inhibitor for Prevention of Colorectal Cancer Recurrence	U	Colorectal Neoplasms	propranolol, etodolac	Rate of recurrent and metastatic cancer. Magnitude and duration of surgically induced immune depression	Etodolac (1600 mg/d) Propranolol (40 mg/d). (1600 mg, day of surgery), (80 - 40 mg/d)	3	400	2010	-
NCT01265576	Study of Sorafenib With or Without VT-122 in Patients With Hepatocellular Carcinoma (HCC)	U	Hepatocellular Carcinoma (HCC)	Sorafenib, VT-122 (propranolol plus etodolac)	Free survival Clinical benefit response	Propranolol (120mg/d) Etodolac (600 mg/d)	2	20	2010	-
NCT01504126	Propranolol Hydrochloride and Chemotherapy in Treating Patients With Ovarian, Primary Peritoneal, or Fallopian Tube Cancer	C	Adenocarcinoma Fallopian Tube Carcinoma Ovarian Clear Cell	propranolol hydrochloride/ chemotherapy	Proportion of patients who successfully complete 6 cycles of chemotherapy with propranolol hydrochloride. Changes in quality of life. Overall survival (OS). Incidence of adverse events. Changes in mood state as measured by Center for	Not provided	1	32	2012	-

					Epidemiologic Studies Depression Scale					
NCT01847001	Study of Propranolol in Newly Diagnosed Breast Cancer Patients Undergoing Neoadjuvant Chemotherapy	C	Locally Advanced Malignant Neoplasm Breast Cancer	paclitaxel nab-paclitaxel trastuzumab pertuzumab doxorubicin cyclophosphamide surgery premedication anti-nausea therapy pegfilgrastim	Percentage Adverse Events. Change in Tumor Proliferation and Density. Changes in Stress Levels	Propranolol (40mg/d), up-titrated to 80mg/day with chemotherapy depending on tolerability. Tolerability is assessed every 2 weeks	2	10	2012	[89]
ACTRN12612 000852853	Preventing Colorectal Cancer Metastases in adults following Surgical Removal of a Primary Tumor by using a cocktail of a B-blocker and a COX2 inhibitor	R	Colorectal cancer	etodolac	Tumor recurrence. Distant metastasis	Not provided	2	206	2012	-
NCT01705392	Bevacizumab vs Dacarbazine in Metastatic Melanoma	T	Metastatic Malignant Melanoma Unresectable Malignant Melanoma	propranolol bevacizumab enalapril dacarbazine	Progression free survival. Response Rates according to RECIST. Disease control rate at 6 months. Prevention of hypertension by beta blockers or ACE-inhibitors. Overall survival	Propranolol (80 mg/d) Bevacizumab (10 mg/kg/d) Enalapril (5 mg/d) Dacarbazine (1000 mg/m²/d)	2	2	2013	-
NCT02013492	Propranolol Hydrochloride in Treating Patients With Locally Recurrent or Metastatic Solid Tumors That Cannot Be Removed By Surgery	ANYR	Male Breast Cancer Melanoma Ovarian Epithelial Cancer Hepatocellular Carcinoma	-	Adverse Events. Changes in VEGF. Tumor microenvironment and host Immune System. Progression-free survival. Overall survival	Not provided	1	1	2014	-
NCT00502684	Perioperative Administration of COX 2 Inhibitors and Beta Blockers to Women Undergoing Breast Cancer Surgery	U	Breast Cancer	etodolac	Levels of NKT cells, lymphocytes, monocytes and granulocytes. *In vitro* levels of cortisol and VEGF	Propranolol (40 mg/d) Etodolac (800 mg/d)	NA	32	2014	[79,80]
NCT02420223	Pilot Study Using Propranolol to Decrease Gene Expression of Stress-Mediated Beta-Adrenergic Pathways in	C	Multiple Myeloma	-	Change in gene expression Change in depression and anxiety Incidence of engraftment syndrome.	Propranolol (40 – 80 mg/d)	2	25	2015	[67,68]

	Hematopoietic Stem Cell Transplant Recipients				Rates of neutrophil and platelet engraftment. Incidence of infection. Differences in myeloma response, mortality, progression-free survival, and overall survival.					
ACTRN12615 000889550	A Phase II randomized study of perioperative beta-blocker vs placebo on gene expression in newly diagnosed breast cancer	C	Breast cancer	-	Primary tumor gene expression Leukocyte gene expression Inflammation-relevant cytokines levels Hypotension, bradycardia. Beck Anxiety Inventory	Propranolol (40 – 80 mg/d)	2	64	2015	[64]
NCT02596867	Neoadjuvant Propanolol in Breast Cancer	T	Breast cancer	-	Tumor size. Proliferative Index. Safety, toxicity and Adherence	Propranolol (3 mg/kg/d)	2	2	2015	[66]
NCT02104011	Treatment of Renal Angiomyolipomas in Tuberous Sclerosis by Beta-blockers	C	Renal Angiomyolipomas Tuberous Sclerosis	-	Evolution of angiomyolipomas volume. Renal function evolution. Effect on the potential haemorraghic transformation. Improvement of the quality of life. Effect on face angiofibromas.	Not provided	2	2	2015	-
NCT02641314	Metronomic Treatment in Children and Adolescents With Recurrent or Progressive High Risk Neuroblastoma	R	Recurrent Neuroblastoma	metronomic therapy	Levels of EFS. Overall survival. Hospitalization days. Number of transfusion days. drop-out rate	Propranolol (0.5 - 2 mg/kg/d) Maximum dose 120 mg/d	2	26	2016	[85]
2011-004593-29	Phase II trial of metronomic treatment in children and adolescents with recurrent or progressive neuroblastoma	O	Neuroblastoma	celecoxib cyclophosphami de etoposide vinblastine	Levels of EFS	Not provided	2	26	2016	-
NCT02962947	MELABLOCK: A Clinical Trial on the Efficacy and Safety of Propranolol 80 mg in Melanoma Patients	NYR	Melanoma	-	Overall survival, free survival, specific mortality, for melanoma patients in stage II/IIIA. Long-term safety on melanoma patients in stage II/IIIA	Propranolol (80 mg/d)	2/3	546	2017	-
NCT03188068	Sirolimus Plus Propranolol Versus Sirolimus Plus	R	Kaposiform Hemangioendothelioma	sirolimus prednisolone	Changes of platelet counts, fibrinogen levels and KHE	Not provided	2	30	2017	-

	Prednisolone for Kaposiform Hemangioendothelioma		Kasabach Merritt Phenomenon		volume.<br>Changes in the patient's symptoms and/or complications.<br>Frequency of adverse events.<br>Change in blood biomarkers.<br>Quality of life in patients					
NCT03384836	Propranolol Hydrochloride and Pembrolizumab in Treating Patients With Stage IIIC-IV Melanoma That Cannot Be Removed by Surgery	R	Cutaneous Melanoma	pembrolizumab	Dose limiting toxicities.<br>Overall response rate and survival.<br>Progression free survival	Not provided	1/2	47	2018	-
NCT03245554	Clinical Research on Treatment of Gastrointestinal Cancer in the Preoperative by Propranolol	U	Gastrointestinal Cancer	-	Tumor size	Not provided	1	80	2018	-
NCT03323710	Study of Propranolol Plus Sunitinib in First-line Treatment of Metastatic Renal Cell Carcinoma	W	Renal Cell Carcinoma	propranolol<br>sunitinib	Objective Response Rate.<br>Overall Survival.<br>Progression-free Survival.<br>Disease Control Rate.<br>Safety profile.<br>Health-related Quality of Life.<br>Disease-related Stress (DRS).<br>Tumour tissue and serum biomarkers status.	Propranolol (80 – 240 mg /d)<br>Sunitinib (50 mg/d) 4 weeks + 2-week rest period cycle	2	0	2018	-
ACTRN12620 000156987	Trial of R-Propranolol to assess the spread of melanoma (MELPROP)	NYR	Malignant melanoma	-	Activation of genes induced by Sox18/RBPJ activity: IL33 and VCAM.	Not provided	1	24	2019	-
NCT03919461	Colorectal Metastasis Prevention International Trial 2	R	Colorectal Neoplasms	etodolac	5-year disease-free-survival.<br>Biomarkers in extracted tumor tissue and in blood samples.<br>Number of patients with treatment related adverse events	Propranolol (40 - 160 mg/d)<br>Etodolac (800 mg/d)	2	200	2019	-
NCT04005365	Clinical Study of Propranolol Combined With Neoadjuvant Chemotherapy in Gastric Cancer	R	Gastric Cancer	neoadjudant chemotherapy	ORR	Not provided	2	78	2019	-
ACTRN12619 001078145	Novel treatment of patients with Advanced Cancers using a combination of commonly available, low-cost oral medications	R	Glioblastoma<br>Metastatic Melanoma<br>Oral cavity Squamous cell carcinoma<br>Head and Neck skin Squamous cell carcinoma	piperine<br>metformin<br>cilazapril<br>losarten	Change in quality of life.<br>Slowing the progression of cancers	Propranolol (80mg/d)<br>Aliskerin (150 mg/d)<br>Aspirin (100 mg/d)<br>Celecoxib (200 mg/d)<br>Curcumin - Piperine (2000 mg/d)<br>Omeprazole (20mg/d)	2	100	2019	-

						Metformin (250 – 1000 mg/d)<br>Cilazapril (1.25 – 5 mg/d)<br>Losarten (50 – 150 mg/d)				
NCT03838029	Perioperative Intervention to Reduce Metastatic Processes in Pancreatic Cancer Patients Undergoing Curative Surgery	R	Pancreatic Neoplasms	etodolac	Rate of cancer recurrence.<br>Biomarkers in extracted tumor tissue and in blood samples.<br>Number of patients with treatment related adverse events	Etodolac (800 mg/d)<br>Propranolol (40 - 160 mg/d)	2	210	2019	-
NCT04493489	Propranolol Adjuvant Treatment of Bladder Cancer	NYR	Bladder Cancer	Bacille Calmette-Guerin (BCG)	Two-year recurrence-free survival	Propranolol (30 – 80 mg/d)	2	242	2020	-
NCT04682158	Propranolol With Standard Chemoradiation for Esophageal Adenocarcinoma	NYR	Esophageal Adenocarcinoma	radiation: intensity<br>carboplatin<br>paclitaxel	Occurrence of Adverse Events.<br>Progression Free Survival.<br>Overall Survival	Not provided	2	60	2021	-
NCT04848519	Propranolol Hydrochloride and Pembrolizumab in Patients With Recurrent or Metastatic Urothelial Carcinoma: A Single Center Phase II Trial	R	(Locally or metastatic) Bladder, Renal, Ureter, Pelvis, Urothelial, Carcinoma<br>Stage IV Bladder Cancer AJCC v8<br>Stage IV Renal Pelvis Ureter<br>Stage IV Urethral Cancer	Pembrolizumab	Proportion of subjects with a best overall response of complete or partial response Clopper-Pearson method	Not provided	2	25	2021	-
NCT05106179	The Efficacy and Safety of Beta-blockers Drugs in Adults With Spinal Hemangioma: a Prospective Cohort Study	R	Spinal Tumor<br>Spinal Hemangioma<br>Beta Blocker Toxicity	Atenolol<br>Propranolol	Reducing tumor growth<br>Reducing tumor<br>Life-quality<br>Valid questionnaires approved by EuroQol Organization"	Not provided	4	100	2021	-
NCT05312255	Non-chemotherapeutic Interventions for the Improvement of Quality of Life and Immune Function in Patients With Multiple Myeloma	NYR	Recurrent or not Plasma Cell Myeloma<br>Refractory or smoldering Plasma Cell Myeloma	Behavioral Intervention<br>Propranolol<br>Quality-of-Life Assessment	Changes in immune cell subsets<br>Adherence rate<br>proportion of patients without clinical or serological progression	Not provided	NA	220	2022	-
